# Hepatoprotective effects of methanol extract of *Carissa opaca *leaves on CCl_4_-induced damage in rat

**DOI:** 10.1186/1472-6882-11-48

**Published:** 2011-06-24

**Authors:** Sumaira Sahreen, Muhammad R Khan, Rahmat A Khan

**Affiliations:** 1 Department of Biochemistry, Faculty of Biological Sciences, Quaid-i-Azam University Islamabad, 44000, Pakistan

**Keywords:** *Carissa opaca*, Carbon tetrachloride, Hepatotoxicity, Oxidative stress, Phytochemical analysis

## Abstract

**Background:**

*Carissa opaca *(Apocynaceae) leaves possess antioxidant activity and hepatoprotective effects, and so may provide a possible therapeutic alternative in hepatic disorders. The effect produced by methanolic extract of *Carissa opaca *leaves (MCL) was investigated on CCl_4_-induced liver damages in rat.

**Methods:**

30 rats were divided into five groups of six animals of each, having free access to food and water *ad libitum*. Group I (control) was given olive oil and DMSO, while group II, III and IV were injected intraperitoneally with CCl_4 _(0.5 ml/kg) as a 20% (v/v) solution in olive oil twice a week for 8 weeks. Animals of group II received only CCl_4_. Rats of group III were given MCL intragastrically at a dose of 200 mg/kg bw while that of group IV received silymarin at a dose of 50 mg/kg bw twice a week for 8 weeks. However, animals of group V received MCL only at a dose of 200 mg/kg bw twice a week for 8 weeks. The activities of aspartate transaminase (AST), alanine transaminase (ALT), alkaline phosphatase (ALP), lactate dehydrogenase (LDH) and γ-glutamyltransferase (γ-GT) were determined in serum. Catalase (CAT), peroxidase (POD), superoxide dismutase (SOD), glutathione-S-transferase (GST), glutathione peroxidase (GSH-Px), glutathione reductase (GSR) and quinone reductase (QR) activity was measured in liver homogenates. Lipid peroxidation (thiobarbituric acid reactive substances; TBARS), glutathione (GSH) and hydrogen peroxide (H_2_O_2_) concentration was also assessed in liver homogenates. Phytochemicals in MCL were determined through qualitative and high performance liquid chromatography (HPLC) analysis.

**Results:**

Hepatotoxicity induced with CCl_4 _was evidenced by significant increase in lipid peroxidation (TBARS) and H_2_O_2 _level, serum activities of AST, ALT, ALP, LDH and γ-GT. Level of GSH determined in liver was significantly reduced, as were the activities of antioxidant enzymes; CAT, POD, SOD, GSH-Px, GSR, GST and QR. On cirrhotic animals treated with CCl_4_, histological studies showed centrilobular necrosis and infiltration of lymphocytes. MCL (200 mg/kg bw) and silymarin (50 mg/kg bw) co-treatment prevented all the changes observed with CCl_4_-treated rats. The phytochemical analysis of MCL indicated the presence of flavonoids, tannins, alkaloids, phlobatannins, terpenoids, coumarins, anthraquinones, and cardiac glycosides. Isoquercetin, hyperoside, vitexin, myricetin and kaempherol was determined in MCL.

**Conclusion:**

These results indicate that MCL has a significant protective effect against CCl_4 _induced hepatotoxicity in rat, which may be due to its antioxidant and membrane stabilizing properties.

## Background

*Carissa opaca *Stapf ex Haines is an evergreen shrub native to the drier parts of Pakistan and India (Himalayas up to 6000 ft), Burma and Sri Lanka [1]. Stems are branched growing up to 3.5 m in height. The traditional knowledge has been suggested as being of special interest as hepatoprotector [2]. The decoction of its bark and leaves is used in disorders related to respiratory dysfunction such as asthma [3]. In Pakistan fruits and leaves are used as an alternative in cardiac disorders [3,4]. This plant possesses antipyretic, aperients activities and is also used in the treatment of cough [5].

Free radicals induce an oxidative state that can lead to cellular membrane injury with the consequent alteration in metabolic processes. Reactive oxygen species (ROS) plays an important role in the pathogenesis of various degenerative human diseases and have been implicated in atherosclerosis, liver disorders, lung and kidney damage, aging and diabetes mellitus [6]. In liver disorders the ability of natural antioxidant system is impaired. Free radicals are generated in cells by environmental factors such as ultraviolet radiation, pollutants, x-rays, as well as by normal metabolism. Carbon tetrachloride (CCl_4_) is a well known hepatotoxin used in diverse experimental models [6]. Liver injuries induced by CCl_4 _are mediated through the formation of reactive intermediates such as trichloromethyl radical (CCl_3 _•) and its derivative trichloromethyl peroxy radical (CCl_3 _OO•), generated by cytochrome P450 of liver microsomes. These free radicals are thought to react with membrane lipids leading to their peroxidation [6]. Membrane disintegration of hepatocytes with subsequent release of aspartate transaminase (AST), alanine transaminase (ALT), alkaline phosphatase (ALP), lactate dehydrogenase (LDH) and γ-glutamyltransferase (γ-GT) marker enzymes of hepatotoxicity, centrilobular necrosis and steatosis are some of the consequences of CCl_4_-induced lipid peroxidation [6]. The intracellular concentration of ROS is a consequence of both their production and removal by various endogenous antioxidants including both enzymatic and non enzymatic components [7,8].

Although a wide range of drugs are currently employed in the management of hepatic disorders. However, alternative approach in recent days is the research of medicament from traditional medicinal systems. Inhibition of free radicals is very important in terms of liver pathology. Natural products from the plant kingdom are being investigated as a source of antioxidants as these may have great relevance in the prevention of diseases associated with oxidative stress [7-9].

Despite the favorable ethnopharmacological properties of *Carissa opaca *leaves, protective effects against hepatotoxicity have not previously been explored. *Carissa opaca *leaves may have a protective effect on the deteriorated hepatic function that results from free radicals generated by CCl_4_. To test this hypothesis, the present investigation examined the ability of methanol extract of *Carissa opaca *leaves (MCL) for protection against CCl_4_-induced oxidative stress in liver. Phyto-constituents of MCL were also characterized in this study.

## Methods

### Chemicals

Reduced glutathione (GSH), oxidized glutathione (GSSG), glutathione reductase, gamma-glutamyl p-nitroanilide, glycylglycine, bovine serum albumin (BSA), 1,2-dithio-bis nitro benzoic acid (DTNB), 1-chloro-2,4-dinitrobenzene (CDNB), reduced nicotinamide adenine dinucleotide phosphate (NADPH), CCl_4_, flavine adenine dinucleotide (FAD), glucose-6-phosphate, Tween-20, 2,6-dichlorophenolindophenol (DCPIP), thiobarbituric acid (TBA), picric acid, sodium tungstate, sodium hydroxide, trichloroacetic acid (TCA) and perchloric acid (PCA) were purchased from Sigma Chemicals Co. St. Louis, USA.

### Extract preparation

The plant was collected in March 2009 from the campus of Quaid-i-Azam University Islamabad, identified by its vernacular name and later validated by Dr. Mir Ajab Khan, Department of Plant Sciences, Quaid-i-Azam University, Islamabad. A voucher specimen was deposited at the Herbarium of Pakistan, Museum of Natural History, Islamabad. Leaves were shade dried for two weeks and powdered in a Willy Mill to 60-mesh size. Briefly, 1 kg powder was extracted with 6 litres of methanol (95%) at 25°C for 48 h. After extraction the mixture was filtered and the methanol solution was evaporated in a rotary evaporator (Panchun Scientific Co., Kaohsiung, Taiwan) at 40°C and stored at 4°C for further *in vivo *investigations.

### Estimation of MCL dose

Male Sprague-Dawley rats (3; six week old) were kept fasting for overnight providing only water, after which the extract was administered intragastrically at the dose of 300 mg/kg bw and rats were remained under observation for 14 days to observe the mortality. Toxicity was not observed, and the procedure was repeated for next higher doses, i.e., 600, 1000, 1500 and 2000 mg/kg bw. One-tenth (200 mg/kg bw) of the maximum dose of the extract tested (2000 mg/kg bw), did not indicate mortality was selected for evaluation of hepatoprotective activity [10].

### Animals and treatment

Six-week-old male Sprague-Dawley rats weighing 180 ± 10 g were kept at 20-22°C on a 12-h light-dark cycle during which time they had free access to standard laboratory rat chow containing protein, 21.05%; fat 4.33%; fiber, 3.07%; ash, 8.3%; sand (silica), 1.65%; carbohydrate, 52.06%; calcium, 0.9%, phosphorous, 0.5%, moisture, 10% (w/w) and fresh water *ad libitum*. We used only male rats because of their constant metabolism compared to the variation in the female physiology. All experimental procedures involving animals were conducted in accordance with the guidelines of National Institute of Health (NIH guidelines Islamabad, Pakistan). The study protocols were approved by Ethical Committee of Quaid-i-Azam University, Islamabad. The rats were acclimatized to laboratory conditions for 7 days before commencement of experiment.

For subchronic toxicity studies, 8 week experiment was designed with some modifications [11]. 30 male rats of Sprague-Dawley strain were randomly divided into five groups with six animals in each. Group (I) the control received only vehicles; olive oil (0.5 ml/kg bw) and DMSO (0.5 ml/kg bw) and fed with a normal diet. Group (II) the CCl_4 _group received intraperitoneal administration of 0.5 ml CCl_4_/kg bw (20% CCl_4_/olive oil) twice a week for 8 weeks to cause subchronic reversible cirrhosis. Group (III) MCL group (200 mg/kg bw) and Group (IV) the silymarin group (50 mg/kg bw) was given intragastrically through a feeding tube twice a week for 8 weeks. These groups (III and IV) also received intraperitoneal injection of 0.5 ml CCl_4_/kg bw (20% CCl_4_/olive oil) twice a week for 8 weeks. Group (V) received only MCL (200 mg/kg bw) twice a week for 8 weeks. At the end of 8 weeks, 24 h of the last treatment, all the animals were anesthesized in an ether chamber. The liver was removed after perfusion with ice cold saline at 4°C. Blood was collected by cardiac puncture and serum obtained by blood centrifugation at 1500 × g for 10 min, at 4°C.

### Assessment of serum marker enzymes

Serum analysis of various liver marker enzymes such as alanine transaminase (ALT), aspartate transaminase (AST), alkaline phosphatase (ALP), lactate dehydrogenase (LDH) and gamma glutamyltransferase (γ-GT) were estimated by using standard AMP diagnostic kits (Stattogger Strasse 31b 8045 Graz, Austria).

### Assessment of liver antioxidant enzymes

10% homogenate of liver tissue was prepared in 100 mM KH_2_PO_4 _buffer containing 1 mM EDTA (pH 7.4) and centrifuged at 12,000 × g for 30 min at 4°C. The supernatant was collected and used for the following experiments as described below. Concentration of protein in supernatant was estimated by using crystalline BSA as standard [12].

#### Catalase assay (CAT)

CAT activity was determined by spectrophotometric method [13]. Briefly, a reaction mixture of 3 ml containing 2.5 ml of 50 mM phosphate buffer (pH 5.0), 0.4 ml of 5.9 mM H_2_O_2 _and 0.1 ml liver supernatant was allowed to react for one min and change in absorbance of the reaction solution was noted at 240 nm. CAT activity was defined an absorbance change of 0.01 as unit/min.

#### Peroxidase assay (POD)

POD activity in the liver supernatant was determined by spectrophotometric method [13]. Briefly, 0.1 ml liver supernatant was added to a reaction mixture containing 2.5 ml of 50 mM phosphate buffer (pH 5.0), 0.1 ml of 20 mM guaiacol and 0.3 ml of 40 mM H_2_O_2_. Change in absorbance of the reaction solution at 470 nm was determined after one min and POD activity was defined an absorbance change of 0.01 as unit/min [13].

#### Superoxide dismutase assay (SOD)

SOD activity was estimated by using NADH as substrate [14]. Briefly, 0.3 ml of supernatant after centrifugation (1500 × g for 10 min followed by 10000 × g for 15 min) was added to the reaction mixture containing 0.1 ml of phenazine methosulphate (186 μM), and 1.2 ml of sodium pyrophosphate buffer (0.052 mM; pH 7.0). Enzyme reaction was initiated by adding 0.2 ml of NADH (780 μM) and stopped after 1 min by adding 1 ml of glacial acetic acid. Amount of chromogen formed was measured by recording color intensity at 560 nm. Results are expressed in units/mg protein.

#### Glutathione-S-transferase assay (GST)

The assay mixture was made using 1.475 ml phosphate buffer (0.1 M, pH 6.5), 0.2 ml reduced glutathione (1 mM), 0.025 ml 1-chloro-2,4-dinitrobenzene (CDNB, 1 mM) and 0.3 ml of liver supernatant. Change in the absorbance was recorded at 340 nm and enzyme activity was calculated as nM CDNB conjugate formed/min/mg protein using a molar extinction coefficient of 9.6 × 10^3^M^-1^cm^-1^[15].

#### Glutathione reductase assay (GSR)

Glutathione reductase assay was carried out by using NADPH as the substrate [16]. Briefly, 0.1 ml of liver supernatant was mixed with 1.65 ml phosphate buffer: (0.1 M; pH 7.6), 0.1 ml EDTA (0.5 mM), 0.05 ml oxidized glutathione (1 mM) and 0.1 ml of NADPH (0.1 mM) and the mixture was measured at 340 nm. Enzyme activity was calculated as nM NADPH oxidized/min/mg protein using molar extinction coefficient of 6.22 × 10^3 ^M^-1^cm^-1^.

#### Glutathione peroxidase assay (GSH-Px)

This procedure was based on a previously reported method [17]. The reaction assay was done in a final volume of 2 ml solution constituted by: 1.49 ml phosphate buffer (0.1 M; pH 7.4), 0.1 ml EDTA (1 mM), 0.1 ml sodium azide (1 mM), 0.05 ml glutathione reductase (1 IU/ml), 0.05 ml GSH (1 mM), 0.1 ml NADPH (0.2 mM), 0.01 ml H_2_O_2 _(0.25 mM) and 0.1 ml of liver supernatant. The disappearance of NADPH at 340 nm was recorded at 25°C. Enzyme activity was calculated as nM NADPH oxidized/min/mg protein using molar extinction coefficient of 6.22 × 10^3 ^M^-1^cm^-1^.

#### Quinone reductase assay (QR)

This procedure was based on the reduction of dichlorophenolindophenol (DCPIP). Briefly, 3.0 ml reaction assay was constituted of 2.13 ml Tris-HCl buffer (25 mM; pH 7.4), 0.7 ml BSA, 0.1 ml FAD, 0.02 ml NADPH (0.1 mM), and 0.1 ml of liver supernatant. The absorbance was determined at 600 nm and enzyme activity was calculated as nM of DCPIP reduced/min/mg protein using molar extinction coefficient of 2.1 × 10^4 ^M^-1^cm^-1^[18].

### Reduced glutathione assay (GSH)

Reduced glutathione in liver homogenate was determined by reaction with 1,2-dithio-bis nitro benzoic acid (DTNB). Briefly, 1.0 ml of supernatant was precipitated with 1.0 ml of (4%) sulfosalicylic acid. The samples were kept at 4°C for 1 h and then centrifuged at 1200 × g for 20 min at 4°C. The total volume of 3.0 ml assay mixture contained 0.1 ml filtered aliquot, 2.7 ml phosphate buffer (0.1 M; pH 7.4) and 0.2 ml of 1,2-dithio-bis nitro benzoic acid (DTNB, 100 mM). The yellow color developed was read immediately at 412 nm on a SmartSpecTM plus Spectrophotometer. It was expressed as μM GSH/g tissue [19].

### Estimation of lipid peroxidation assay (TBARS)

Malondialdehyde in liver homogenate was determined by reaction with thiobarbituric acid (TBA). Briefly, 1.0 ml reaction assay was consisted of 0.58 ml phosphate buffer (0.1 M; pH 7.4), 0.2 ml liver supernatant, 0.2 ml ascorbic acid (100 mM), and 0.02 ml ferric chloride (100 mM). The reaction mixture was incubated at 37°C in a shaking water bath for 1 h. The reaction was stopped by addition of 1.0 ml of trichloroacetic acid (10%). Following addition of 1.0 ml 0.67% thiobarbituric acid, all the tubes were placed in boiling water bath for 20 min and then shifted to crushed ice-bath before centrifuging at 2500 × g for 10 min. The amount of TBARS formed in each of the samples was assessed by measuring optical density of the supernatant at 535 nm using spectrophotometer against a reagent blank. The results were expressed as nM TBARS/min/mg tissue at 37°C using molar extinction coefficient of 1.56 ×10^5 ^M^-1^cm^-1^[20].

### Hydrogen peroxide assay (H_2_O_2_)

Hydrogen peroxide (H_2_O_2_) was assayed by H_2_O_2_-mediated horseradish peroxidase-dependent oxidation of phenol red [21]. 2.0 ml of liver supernatant was suspended in 1.0 ml of solution containing phenol red (0.28 nM), horse radish peroxidase (8.5 units), dextrose (5.5 nM) and phosphate buffer (0.05 M; pH 7.0) and the resultant mixture was incubated at 37°C for 60 min. The reaction was stopped by the addition of 0.01 ml of NaOH (10 N) and then centrifuged at 800 × g for 5 min. The absorbance of the supernatant was recorded at 610 nm against a reagent blank. The quantity of H_2_O_2 _produced was expressed as nM H_2_O_2_/min/mg tissue based on the standard curve of H_2_O_2 _oxidized phenol red.

### Histopathological studies

For microscopic evaluation liver tissues were fixed in a fixative (absolute alcohol 60%, formaldehyde 30%, glacial acetic acid 10%) and embedded in paraffin, sectioned at 4 μm and subsequently stained with hematoxylin/eosin. First the slides were deparaffinized in xylene, and rehydrated in descending order of ethanol. Slides were dipped in hematoxylin, washed in tap water and dehydrated in ascending order of ethanol. Sildes were then stained with eosin and washed with absolute ethanol and xylene. Sections were studied under light microscope (DIALUX 20 EB) at 40× magnifications.

### Phytochemical screening

Qualitative tests of the MCL for the presence of alkaloids, anthraquinones, cardiac glycosides, coumarins, flavonoids, saponins, phlobatannins, tannins and terpenoids were carried out.

#### Test for alkaloids

0.4 g of MCL was stirred with 8 ml of 1% HCl, mixture was warmed and filtered. 2 ml of filtrate was treated separately with (a) with few drops of potassium mercuric iodide (Mayer's reagent) and (b) potassium bismuth (Dragendroff's reagent). Turbidity or precipitation with either of these reagents was taken as evidence for existence of alkaloids [22].

#### Test for saponins

The ability of saponins to produce emulsion with oil was used for the screening test. 20 mg of MCL was boiled in 20 ml of distilled water in a water bath for five min and filtered. 10 ml of the filtrate was mixed with 5 ml of distilled water and shaken vigorously for froth formation. 3 drops of olive oil were mixed with froth, shaken vigorously and observed for emulsion development [22].

#### Test for terpenoids

Presence of terpenoids in MCL was carried out by taking 5 ml (1 mg/ml) of MCL and mixed with 2 ml of chloroform, followed by 3 ml of concentrated H_2_SO_4_. A reddish brown coloration of the interface confirmed the presence of terpenoids [22].

#### Test for anthraquinones

200 mg of MCL was boiled with 6 ml of 1% HCl and filtered. The filtrate was shaken with 5 ml of benzene, filtered and 2 ml of 10% ammonia solution was added to the filtrate. The mixture was shaken and the presence of a pink, violet or red color in the ammoniacal phase indicated the presence of free hydroxy anthraquinones [23].

#### Cardiac glycosides determination

5 ml (10 mg/ml in methanol) of MCL was mixed with 2 ml of glacial acetic acid having one drop of FeCl_3 _solution. To the mixture obtained 1 ml of concentrated H_2_SO_4 _was added to form a layer. The presence of brown ring of the interface indicated deoxy sugar characteristic of cardiac glycosides [23].

#### Test for coumarins

In a small test tube, 300 mg of MCL was covered with filter paper moistened with 1 N NaOH. The test tube was placed for few minutes in a boiling water bath. After removing the filter paper it was examined under UV light, yellow florescence indicated the presence of coumarins [23].

#### Test for phlobatannins

80 mg of MCL was boiled in 1% aqueous hydrochloric acid; the deposition of a red precipitate indicated the presence of phlobatannins [23].

#### Test for flavonoids

*Test for *: 50 mg of MCL was suspended in 100 ml of distilled water to get the filtrate. 5 ml of dilute ammonia solution was added to 10 ml of filtrate followed by few drops of concentrated H_2_SO_4_. Presence of flavonoids was confirmed by yellow coloration [24].

#### Test for tannins

50 mg of MCL was boiled in 20 ml of distilled water and filtered. A few drops of 0.1% FeCl_3 _was added in filtrate and observed for color change; brownish green or a blue-black coloration was taken as evidence for the presence of tannins [24].

#### High Performance Liquid Chromatography (HPLC) of MCL

*Sample preparation*: 50 mg of MCL was extracted with 6 ml of 25% hydrochloric acid and 20 ml methanol for 1 h. The obtained extract was filtered to a volumetric flask. The residue was heated twice with 20 ml of methanol for 20 min to obtain the extract. The combined extract was diluted with methanol to 100 ml. 5 ml portion of the solution was filtered and transferred to a volumetric flask and diluted with 10 ml of methanol. The sample (10 μl) was injected into the HPLC apparatus.

*HPLC determination*: Samples were analyzed on Agilent HPLC system. Separation was carried out through column 20RBAX ECLIPSE, XDB-C18, (5 μm; 4.6 × 150 mm, Agilent USA) with UV-VIS Spectra-Focus detector, injector-auto sampler. Solvent A (0.05% trifluoroacetic acid) and solvent B (0.038% trifluoroacetic acid in 83% acetonitrile (v/v) with the following gradient: 0-5 min, 15% B in A, 5-10 min, 50% B in A, 10-15 min, 70% B in A. The flow rate was 1 ml/min and injection volume was 10 μl. Eleven standard compounds including rutin, myricetin, vitexin, orientin, hyperoside, isovitexin, isoquercetin, luteolin, apigenin, kaempherol, and luteolin-7-glucoside were run for comparative detection and were optimized. The calibration curves were defined for each compound in the range of sample quantity 0.02-0.5 μg. All samples were assayed in triplicate.

### Statistical analysis

The values are expressed as means ± standard deviation (SD) of six observations in each group. One-way analysis of variance (ANOVA) was carried out to determine the significant difference between parameters. Duncan's multiple range test was used to determine the significant difference between the groups at P < 0.05 by using SPSS ver. 14.0.

## Results

### Chemical composition of MCL

#### Qualitative analysis of MCL

Phytochemical analysis of MCL demonstrated the presence of alkaloids, anthroquinones, cardiac glycosides, coumarins, flavonoids, saponins, phlobatannins, tannins and terpenoids.

#### HPLC analysis of MCL

**Figure 1:**
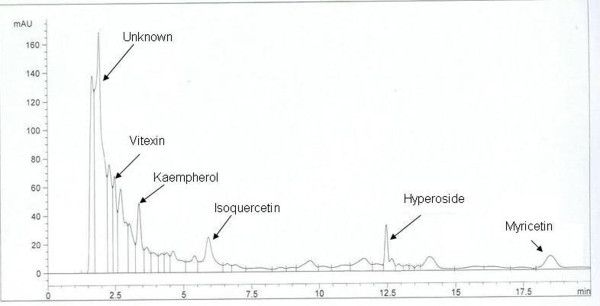
**HPLC flavonoids profile of MLC**.

HPLC analysis of MCL revealed the presence of chromatographic peaks consistent with the pattern showed by the standards such as isoquercetin, hyperoside, vitexin, myricetin and kaempherol. Quantitative HPLC analysis showed that myricetin (0.172 μg/mg of MCL) and isoquercetin (0.119 μg/mg of MCL) were the main flavonoids in MCL (Table 1; Figure 1).

**Table 1:** Flavonoids profile of MCL through HPLC studies

Compound	Retention time (min)	Concentration μg/mg of MCL dry weight
Unknown	2	0.067 ± 0.008
Isoquercetin	6	0.119 ± 0.006
Hyperoside	12.5	0.062 ± 0.005
Vitexin	2.5	0.053 ± 0.004
Myricetin	18.5	0.172 ± 0.009
Kaempherol	3.4	0.084 ± 0.015

MCL; methanol extract of *Carissa opaca *leaves

Values are Mean ± SD (03 number)

### Hepatoprotective studies

#### Effects of MCL on serum marker enzymes

Blood biochemical parameters such as ALT, AST, ALP, LDH and γ-GT level, of all the experimental groups are shown in Table 2. CCl_4 _induced an increase in enzymatic activity of ALT, AST, ALP, LDH and γ-GT as compared to the control group. However co-treatment with MCL (200 mg/kg bw) reduced the alterations in the activity level of ALT, AST, ALP, LDH and γ-GT as against the CCl_4 _group. Silymarin (50 mg/kg bw) prevented the alterations in the activity level of ALT, AST, ALP, LDH and γ-GT induced with CCl_4_.

**Table 2:** Effects of MCL on liver function tests in rat

Group	AST(U/l)	ALT(U/l)	ALP(U/l)	γ-GT(U/l)	LDH (U/l)
I	104.12 ± 8.2+	69.9 ± 6.8+	146.34 ± 14.2+	2.01 ± 0.86+	46.7 ± 4.16+
II	211.56 ± 18.3*	221.6 ± 24.7*	315.67 ± 19.2*	4.61 ± 0.48*	133.9 ± 14.12*
III	111.61 ± 8.2*+	85.6 ± 6.8*+	188.39 ± 14.8*+	2.39 ± 0.65*+	61.8 ± 6.27*+
IV	123.76 ± 9.4*+	87.9 ± 7.7*+	201.45 ± 16.8*+	2.69 ± 0.79*+	64.32 ± 7.82*+
V	101.24 ± 8.5+	74.6 ± 6.6+	152.54 ± 16.4+	1.92 ± 0.53+	38.65 ± 3.44+

Group I: Control (Olive oil+ DMSO); II: CCl_4 _(0.5 ml/kg bw); III: MCL (200 mg/kg bw)+CCl_4 _(0.5 ml/kg bw); IV: Silymarin (50 mg/kg bw)+CCl_4 _(0.5 ml/kg bw); V: MCL (200 mg/kg bw).

MCL; methanol extract of *Carissa opaca *leaves

Values are Mean ± SD (06 number)

*, Indicate significance from control group (P < 0.05)

+, Indicate significance from CCl_4 _group (P < 0.05)

#### Effects of MCL on liver enzymatic antioxidant level

CCl_4 _decreased the activities of hepatic antioxidant enzymes; CAT, SOD, POD, GSR, GSH-Px, QR and GST as compared to the respective control group_. _Co-administration of MCL and silymarin avoided the CCl_4_-induced decrease in activities of hepatic antioxidant enzymes (Table 3).

**Table 3:** Effects of MCL on hepatic antioxidant profile in rat

Group	CAT (U/min)	POD (U/min)	SOD (U/mg protein)	GST (μM CDNB conjugate/min/mg protein)	GSH-Px (nM of NADPH/min/mg protein)	GSR (nM of NADPH/min/mg protein)	QR (nM of DCPIP reduced/min/mg protein)
I	4.59 ± 0.57+	10.34 ± 0.67+	3.62 ± 0.23+^e^	167.89 ± 9.67+	125.91 ± 5.12+	220.78 ± 10.96+	108.53 ± 6.06+
II	2.15 ± 0.74*	5.23 ± 0.45*	0.98 ± 0.09*	98.76 ± 7.45*	70.84 ± 4.68*	134.74 ± 9.72*	58.60 ± 3.53*
III	4.12 ± 0.35*+	9.87 ± 0.52*+	3.11 ± 0.14*+	150.78 ± 6.99*+	112.79 ± 5.02*+	194.23 ± 8.90*+	89.80 ± 4.08*+
IV	3.99 ± 0.64*+	9.34 ± 0.78*+	2.98 ± 0.04*+	154.23 ± 7.83*+	110.56 ± 4.81*+	189.48 ± 12.49*+	89.24 ± 5.21*+
V	4.62 ± 0.26+	10.52 ± 0.67+	3.65 ± 0.16+	168.25 ± 6.48+	122.34 ± 4.67+	223.56 ± 7.68+	110.43 ± 4.57+

Group I: Control (Olive oil+ DMSO); II: CCl_4 _(0.5 ml/kg bw); III: MCL (200 mg/kg bw)+CCl_4 _(0.5 ml/kg bw); IV: Silymarin (50 mg/kg bw)+CCl_4 _(0.5 ml/kg bw); V: MCL (200 mg/kg bw).

MCL; methanol extract of *Carissa opaca *leaves

Values are Mean ± SD (06 number)

*, Indicate significance from control group (P < 0.05)

+, Indicate significance from CCl_4 _group (P < 0.05)

#### Effects of MCL on liver protein, glutathione, H_2_O_2 _and lipid peroxidation

Table 4 depicts the effects of MCL on proteins, GSH, TBARS and H_2_O_2 _levels. CCl_4 _treatment significantly increased the hepatic TBARS and H_2_O_2 _level whereas significantly decreased the GSH and protein contents than that of the control group. MCL and silymarin prevented the CCl_4_-induced alterations in TBARS, H_2_O_2_, GSH and protein of liver homogenates.

**Table 4:** Effects of MCL on hepatic protein, lipid peroxidation (TBARS), glutathione (GSH) and hydrogen peroxide (H_2_O_2_)

Group	Protein (μg/mg tissue)	TBARS (nM/mg protein)	GSH (μg/mg protein)	H_2_O_2 _(nM H_2_O_2_/min/mg tissue)
I	1.598 ± 0.034+	3.67 ± 0.53+	21.34 ± 1.10+	1.594 ± 0.016+
II	0.976 ± 0.023*	6.21 ± 0.98*	11.56 ± 1.67*	2.984 ± 0.077*
III	1.501 ± 0.081*+	4.32 ± 0.91*+	17.67 ± 1.16*+	1.710 ± 0.067*+
IV	1.492 ± 0.009*+	4.51 ± 0.74*+	18.48 ± 1.21*+	1.640 ± 0.053*+
V	1.685 ± 0.014+	3.53 ± 0.42+	20.68 ± 1.12+	1.521 ± 0.043+

Group I: Control (Olive oil+ DMSO); II: CCl_4 _(0.5 ml/kg bw); III: MCL (200 mg/kg bw)+CCl_4 _(0.5 ml/kg bw); IV: Silymarin (50 mg/kg bw)+CCl_4 _(0.5 ml/kg bw); V: MCL (200 mg/kg bw).

MCL; methanol extract of *Carissa opaca *leaves

Values are Mean ± SD (06 number)

*, Indicate significance from control group (P < 0.05)

+, Indicate significance from CCl_4 _group (P < 0.05)

## Discussion

In the present investigation administration of CCl_4 _to rats was shown to cause oxidative stress in liver and this damage was associated with significantly lowered activities of antioxidant enzymes; CAT, POD, SOD, GSH-Px, GSR, GST and QR. Concentration of H_2_O_2 _was significantly increased in liver samples with the administration of CCl_4 _to rats. On cirrhotic animals treated with CCl_4_, a significant increase in lipid peroxides while a significant decrease in glutathione in liver samples indicated the severity of CCl_4 _to rats. Co-treatment of MCL, on the other hand, prevented the toxic effects of CCl_4 _by restoring the activities of antioxidant enzymes and glutathione towards the level of control animals. It seems likely that CCl_4 _administration cause oxidative stress in liver via the generation of free radicals whereas the MCL ameliorates the liver injuries by scavenging of free radicals, which is further confirmed by the reduced amount of histopathological injury [10,25,26].

**Figure 2:**
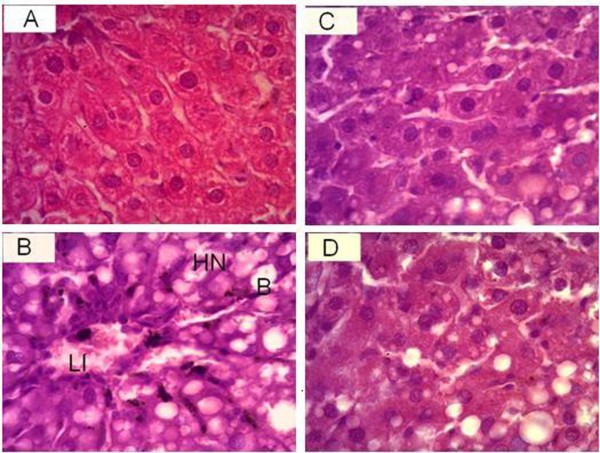
**Micrograph of liver of rats (H & E stain)**. (A) Representative section of liver from the control group showing the normal histology. (B) CCl_4 _(0.5 ml/kg bw) induced hydropic necrosis (HN), lymphocytes infiltration (LI), and ballooning (B) of hepatocytes. (C) CCl_4_+MCL (200 mg/kg bw) repairing of hepatocytes. (D) CCl_4_+silymarin (50 mg/kg bw) repairing of hepatocytes.

Lipid peroxidation is supposed to be a critical factor in the pathogenesis of CCl_4_-induced hepatic injuries. In this study, rats treated with CCl_4 _developed significant hepatic damage as manifested by a significant increase in activities of AST, ALT, ALP, LDH and γ-GT that are indicators of hepatocyte damage and loss of functional integrity. Centrilobular necrosis, lymphocytes infiltration and steatosis were also apparent in this study (Figure 2) [27]. Phosphatases are key enzymes responsible for various biological functions such as metabolism, detoxification and biosynthesis of energetic macromolecules for different essential functions. Any alteration in the activity of these enzymes causes tissue lesion and cellular impairment and dysfunction. Previous studies have reported that oxidative stress plays an essential role in the hepatic injury mediated by CCl_4 _[28-30]. Decrease in the level of these enzymes with MCL is an indication of the stabilization of plasma membrane as well as repair of liver damage caused by CCl_4_, and is similar to earlier reports [7,9].

The coordinate action of antioxidant system is very critical for the detoxification of free radicals. SOD reduces the concentration of highly reactive superoxide radical by converting it to H_2_O_2 _whereas CAT and GSH-Px decomposes H_2_O_2 _and protect the tissues from highly reactive hydroxyl radicals. In the present study, MCL alleviated the CCl_4_-mediated oxidative stress with an increase in activities of antioxidant enzymes; CAT, POD, SOD, GSH-Px, GSR, GST and QR in hepatic samples. Previous studies have reported that CCl_4 _reduces the activities of antioxidant enzymes and causes hepatopathy [28-31]. Interestingly, impaired hepatic antioxidant enzymes activities were brought back to increase by the treatment of MCL to CCl_4_-treated rats. Thus, the antioxidant activity or the inhibition of generation of free radicals by MCL is important in the protection against CCl_4_-induced hepatopathy [32].

GSH is supposed to be a highly effective extra-and intra-cellular antioxidant compound that neutralizes hydrogen peroxide and hydroperoxides by its scavenging and antioxidant properties. In this study, CCl_4 _treatment to rats depletes the hepatic GSH contents. A number of studies have revealed that GSH conjugates play a major role in eliminating the CCl_4_-induced toxic metabolites which are the main cause of liver injuries. The maintenance of sufficient glutathione level is important for the prevention of CCl_4_-induced damages [33]. Toxicants that deplete GSH or influence the activity of GSH-dependent enzyme(s) may result in toxic responses. The study herein presented was instigated by other reports where CCl_4 _administration cause depletion in GSH contents [34,35]. The mechanism of hepatoprotection by MCL against the CCl_4 _toxicity might be due to restoration of GSH concentration in liver as reported previously [34,35].

An approach for the detection of hepatic injury involves measurement of lipid peroxides. The observed decline in lipid peroxides in liver samples of rat following the co-treatment with CCl_4 _and MCL suggests the protective potential of MCL by scavenging of free radicals produced by CCl_4 _(34,35). We obtained significant increase in H_2_O_2 _concentration with CCl_4 _in liver samples of rat as compared to the control group in this experiment. The oxidative damage observed in hepatic samples of rat with CCl_4 _treatment could be the consequence of hydrogen abstraction from membrane lipid molecules by H_2_O_2_-derived OH• and the failure of antioxidants to reestablish redox homeostasis [36,37]. Lipid peroxidation leads to a cascade of reactions, thereby not only destroys membrane lipids but also generates endogenous toxicants that can readily react with adjacent molecules like membrane proteins or diffuse to more distant molecules like DNA, which may lead to more hepatic complications and functional anomalies.

We hypothesize that MCL would be able to protect CCl_4_-induced hepatic damages in rat liver as an intrinsic ameliorating properties. Phyto-constituents such as saponins, tannins, terpenoids and flavonoids (isoquercetin, hyperoside, vitexin, myricetin and kaempherol) present in MCL; have been reported to exert antioxidant activities by scavenging of free radicals that cause lipid peroxidation [9,25].

## Conclusion

On the whole, it can be concluded that the altered biochemical profiles due to CCl_4 _exposure is reversed towards normalization by MCL. The contents of the extract not only increased the regenerative and reparative capacity of the liver but, at the same time prevented from oxidative damage. Beneficial effects of MCL illustrated in this study may be due to the presence of phyto-components that have membrane stabilizing effects.

## Competing interests

The authors declare that they have no competing interests.

## Authors' contributions

SS made significant contribution to acquisition of data, analysis, drafting of the manuscript. MRK has made substantial contribution to conception and design, interpretation of data, drafting and revising the manuscript for intellectual content. RAK participated in the design and collection of data and analysis. All authors read and approved the final manuscript.

## Pre-publication history

The pre-publication history for this paper can be accessed here:

http://www.biomedcentral.com/1472-6882/11/48/prepub
